# Methylation and protein expression of DNA repair genes: association with chemotherapy exposure and survival in sporadic ovarian and peritoneal carcinomas

**DOI:** 10.1186/1476-4598-8-48

**Published:** 2009-07-14

**Authors:** Elizabeth M Swisher, Rachel M Gonzalez, Toshiyasu Taniguchi, Rochelle L Garcia, Tom Walsh, Barbara A Goff, Piri Welcsh

**Affiliations:** 1 Division of Gynecologic Oncology, Department of Obstetrics and Gynecology, University of Washington School of Medicine, Seattle, WA 98195, USA; 2 Division of Medical Genetics, Departments of Medicine, University of Washington School of Medicine, Seattle, WA 98195, USA; 3 Divisions of Human Biology and Public Health Sciences, Fred Hutchinson Cancer Research Center, Seattle, WA 98109-1024, USA; 4 Department of Pathology, University of Washington School of Medicine, Seattle, WA 98195, USA

## Abstract

**Background:**

DNA repair genes critically regulate the cellular response to chemotherapy and epigenetic regulation of these genes may be influenced by chemotherapy exposure. Restoration of BRCA1 and BRCA2 mediates resistance to platinum chemotherapy in recurrent BRCA1 and BRCA2 mutated hereditary ovarian carcinomas. We evaluated BRCA1, BRCA2, and MLH1 protein expression in 115 sporadic primary ovarian carcinomas, of which 31 had paired recurrent neoplasms collected after chemotherapy. Additionally, we assessed whether promoter methylation of BRCA1, MLH1 or FANCF influenced response to chemotherapy or explained alterations in protein expression after chemotherapy exposure.

**Results:**

Of 115 primary sporadic ovarian carcinomas, 39 (34%) had low BRCA1 protein and 49 (42%) had low BRCA2 expression. BRCA1 and BRCA2 protein expression were highly concordant (p < 0.0001). MLH1 protein loss occurred in 28/115 (24%) primary neoplasms. BRCA1 protein loss in primary neoplasms was associated with better survival (p = 0.02 Log Rank test) and remained significant after accounting for either stage or age in a multivariate model (p = 0.04, Cox proportional hazards). In paired specimens, BRCA1 protein expression increased in 13/21 (62%) and BRCA2 protein expression increased in 15/21 (71%) of recurrent carcinomas with low or intermediate protein in the paired primary. In contrast MLH1 expression was rarely decreased in recurrent carcinomas (1/33, 3%). Similar frequencies of MLH1, BRCA1, and FANCF promoter methylation occurred in primary carcinomas without previous chemotherapy, after neoadjuvant chemotherapy, or in recurrent neoplasms.

**Conclusion:**

Low BRCA1 expression in primary sporadic ovarian carcinoma is associated with prolonged survival. Recurrent ovarian carcinomas commonly have increased BRCA1 and/or BRCA2 protein expression post chemotherapy exposure which could mediate resistance to platinum based therapies. However, alterations in expression of these proteins after chemotherapy are not commonly mediated by promoter methylation, and other regulatory mechanisms are likely to contribute to these alterations.

## Background

Ovarian carcinoma is the most deadly gynecological malignancy and is the fifth leading cause of carcinoma death in American women. Ovarian carcinomas are usually responsive to initial platinum based chemotherapy regimens. However, even after a complete clinical response, most ovarian carcinomas do recur, with resistance to platinum therapy developing after one or more chemotherapy courses. Acquired chemotherapy resistance is one of the greatest clinical challenges in the treatment of women with ovarian carcinoma.

Germline mutations in the BRCA1 and BRCA2 genes confer inherited susceptibility to ovarian and breast carcinomas. Breast and ovarian carcinomas in BRCA1 or BRCA2 mutation carriers usually have somatic deletions of the wildtype allele, rendering the neoplasm BRCA1 or BRCA2 deficient. BRCA2 is identical to the Fanconi anemia (FA) gene FANCD1[1]. BRCA1, BRCA2, other FA genes, and a larger number of protein partners (including the DNA mismatch repair protein MLH1) are part of a complex DNA damage response network (reviewed in[2]). *In vitro *studies indicate that BRCA1 and BRCA2 loss increases sensitivity to agents that cause double strand DNA breaks and/or interstrand DNA cross-links including platinum agents [3-5]. Conversely, loss of BRCA1 or BRCA2 may increase resistance to microtubule interfering agents such as taxanes and vincristine [6,7]. Most studies report improved survival in women with ovarian carcinomas associated with BRCA1 and BRCA2 mutations compared to women with sporadic ovarian carcinoma, consistent with increased sensitivity to platinum-based chemotherapy [8-10]. We and others have recently shown that carcinomas from patients with inherited frameshift mutations in BRCA1 or BRCA2 exposed to chemotherapy can acquire secondary mutations that restore the reading frame of BRCA1 or BRCA2, resulting in platinum resistance [11-13]. Thus, restoration of expression of proteins in the FA-BRCA DNA damage response pathway may increase resistance to platinum and potentially other agents that induce DNA damage.

While germline mutations in BRCA1 and BRCA2 predispose to hereditary ovarian carcinoma, somatic mutations in these genes are rare in sporadic ovarian carcinomas [14-17]. However, epigenetic alterations in these and other DNA repair genes may play important roles in sporadic ovarian and breast carcinomas and could contribute to responsiveness to chemotherapy. Promoter methylation leads to decreased expression of BRCA1, MLH1, and FANCF protein in a subset of ovarian carcinomas [18-23]. BRCA1 promoter methylation occurs in 5–20% of sporadic ovarian carcinomas[20,22,24,25], while BRCA2 methylation is rare[26,27]. FANCF is a key regulator of the FA-BRCA pathway[23]. Epigenetic regulation of gene transcription through promoter methylation or histone acetylation could be a modifiable mechanism of sensitivity or resistance to chemotherapy. Indeed, *in vitro *data indicate that epigenetic alterations in MLH1 and FANCF modulate response to platinum agents in cell lines [23,28]. While loss of BRCA1, BRCA2, or FANCF confers sensitivity to platinum, loss of MLH1 confers resistance to platinum agents [28]. The importance of these mechanisms in *in vivo *chemoresistance in ovarian carcinomas has not been defined.

We undertook this study to determine if protein expression and/or promoter methylation of BRCA1, MLH1, BRCA2 and FANCF predicted overall survival in primary sporadic ovarian carcinomas and whether promoter methylation or protein expression were altered by exposure to chemotherapy in recurrent carcinomas. We also wanted to determine the relationship of alterations of proteins in the FA-BRCA pathway with alterations in p53, another key sensor of DNA damage.

## Results

### BRCA1, MLH1 and BRCA2 protein expression in primary ovarian carcinomas

Protein expression for BRCA1, MLH1 and BRCA2 was assessed with immunohistochemistry in 115 sporadic primary invasive neoplasms. In 31 cases, we had paired recurrent or persistent neoplasm tissue available after chemotherapy which allowed for comparison of protein expression with the matched primary. Eight primary invasive carcinomas were classified as peritoneal in origin and the remainder ovarian in origin (Table 1). Clinical and pathological characteristics and relationship to BRCA1 protein expression are summarized in Table 1. Peritoneal carcinomas were less likely to have low BRCA1 protein than ovarian carcinomas (p = 0.049, Fishers Exact, two-tailed). There was no association between BRCA1 protein expression and grade, histology, or adequacy of surgical cytoreduction. Stage I carcinomas were more likely to have normal BRCA1 protein expression compared to stage II-IV carcinomas (p = 0.03, Fisher exact, two-tailed). As expected both stage I and II carcinomas were also more likely to have non serous and undifferentiated histologies (8/11, 73% for stage I and 3/4 for stage II, p < 0.0001 compared to stage III and IV, Fishers Exact, two-tailed). Stage I histologies included two serous, one undifferentiated carcinoma, one small cell, two mucinous, five endometrioid, while stage II included 3 endometrioid and one serous. However, normal BRCA1 protein expression was associated with Stage I disease and not histology. Both serous stage I carcinomas had normal BRCA1 protein expression and 2/3 of endometrioid stage II carcinomas had low BRCA1 protein expression.

**Table 1:** Clinicopathological characteristics of the sporadic neoplasms studied and BRCA1 protein expression.

**BRCA1 Protein Expression**					
	**All Cases**	**Low**	**Intermediate**	**Normal**	**p value**
	
Primary site					
Ovarian	107 (93%)	39	27	41	p = 0.05
Peritoneal	8 (7%)	0	3	5	
					
**Grade**					
Grade 1	6 (5%)	3	2	1	
Grade 2	16 (16%)	5	3	8	NS
Grade 3	93 (79%)	31	25	37	
					
**Histology**					
Serous	81 (70%)	28	24	29	NS
Endometrioid	11 (10%)	4	2	5	
Carcinoma NOS	12 (10%)	3	3	6	
MMMT	4 (3%)	1	1	2	
Clear Cell	1 (1%)	0	0	1	
Mucinous	3(3%)	1	0	2	
Other*	3 (3%)	2	0	1	
					
**Stage**					
I	11 (10%)	2	1	8	p = 0.03
II	4 (3%)	3	0	1	
III	83 (72%)	28	26	29	
IV	17 (15%)	6	3	8	
					
**Cytoreduction**					
Optimal (< 1 cm)	73	24	18	31	NS
Suboptimal	36	14	9	13	
Not Available	6	1	3	2	
**Total**	**116**	**39 (36%)**	**30 (26%)**	**46 (38%)**	

* Other histologies included one small cell, one transitional cell carcinoma and one carcinoma with focal giant cells.

**Figure 1:**
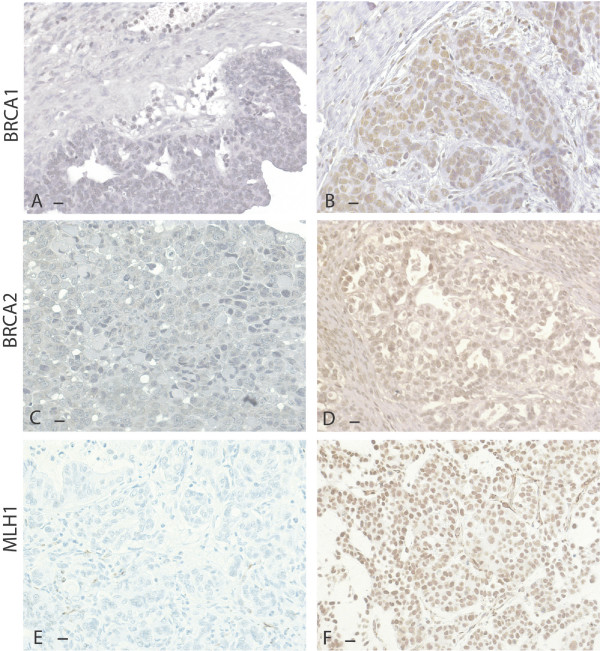
**Representative protein expression of BRCA1, BRCA2 and MLH1 in sporadic ovarian carcinomas**. Protein expression is represented by brown stain. Black bars in the lower left corners represent 10 microns. A. BRCA1 protein in a neoplasm with low expression. B. BRCA1 protein in a neoplasm with normal expression. C. BRCA2 protein in a neoplasm with low expression. D. BRCA2 protein in a neoplasm with normal expression. E. MLH1 protein in a neoplasm with low expression. F. MLH1 protein in a neoplasm with normal expression.

BRCA1, BRCA2, and MLH1 protein expression is summarized in Table 2 and representative staining is shown in Figure 1. BRCA1 and BRCA2 protein levels were significantly and positively correlated (p < 0.0001, two-tailed). BRCA1 and BRCA2 protein expression were not related to MLH1 protein expression.

**Table 2:** Protein expression in primary sporadic ovarian and peritoneal carcinomas before chemotherapy exposure.

	**Protein Expression**			
	**Low** **(≤10%)**	**Intermediate** **(10–30%)**	**Normal** **(>30%)**	**Total**
BRCA1 expression	39 (34%)	30 (26%)	46 (40%)	115
BRCA2 expression	49 (42%)	28 (24%)	38 (33%)	115
MLH1 Expression*	28 (24%)		87 (76%)*	115

MLH1 expression was scored in two categories: ≤10% positive cells = Low, >10% positivity = Normal.

### BRCA1, MLH1 and BRCA2 protein expression in paired primary and recurrent ovarian carcinomas

**Figure 2:**
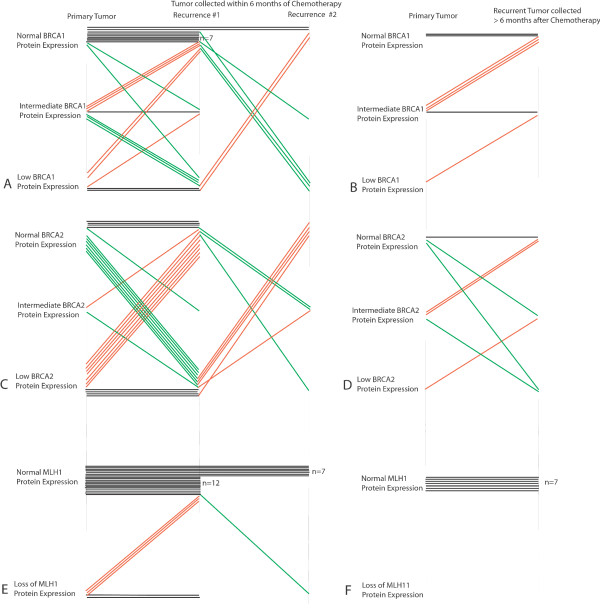
**Schematic of BRCA1, BRCA2, and MLH1 protein expression in paired primary and recurrent neoplasms**. Each neoplasm is represented by a single horizontal line. A. BRCA1 protein expression in 24 primary and paired recurrent neoplasms obtained ≤ 6 months since last chemotherapy, 8 with second recurrences. B. BRCA1 expression in 7 primary and paired recurrent neoplasms in which the recurrence was obtained more than 6 months since last chemotherapy. C. BRCA2 protein expression in 24 primary and paired recurrent neoplasms obtained ≤ 6 months since last chemotherapy. D. BRCA2 expression in 7 primary and paired recurrent neoplasm obtained more than 6 months since last chemotherapy. E. MLH1 protein expression in 24 primary and paired recurrent neoplasms obtained ≤ 6 months since last chemotherapy. F. MLH1 expression in 7 primary and paired recurrent neoplasm obtained more than 6 months since last chemotherapy.

**Figure 3:**
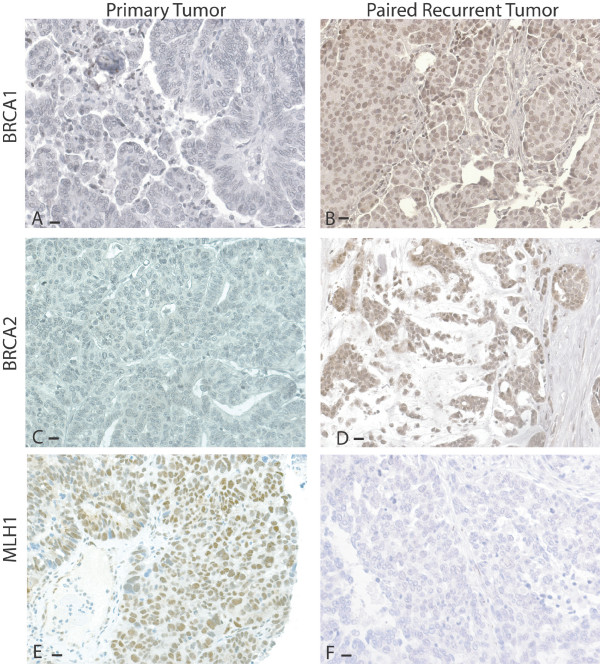
**BRCA1, BRCA2, and MLH1 protein expression in paired primary and recurrent neoplasms**. Protein expression is represented by brown stain. Black bars in the lower left corners are equal to 10 microns. A. BRCA1 expression is low in the primary neoplasm. B. BRCA1 expression is increased in the paired recurrent neoplasm. C. BRCA2 protein expression is low in a different primary neoplasm. D. BRCA2 expression is increased in the paired recurrent neoplasm. E. MLH1 protein is normal in a different primary neoplasm. F. In the paired recurrent neoplasm, MLH1 protein expression is reduced.

MLH1, BRCA1, and BRCA2 protein expression was also assessed in 31 matched primary and recurrent ovarian carcinomas from the same patient to determine if chemotherapy influenced MLH1, BRCA1, or BRCA2 protein levels. In 7 cases a second recurrence was available for expression analyses. For second recurrences, data was tabulated as if for a separate case when compared to the primary. Recurrent neoplasms were obtained at varying time intervals from last chemotherapy. We separated matched pairs into two groups depending on the interval since last chemotherapy exposure (≤ six months or >six months). Complete data for protein and methylation analyses for each case is presented in Table S1; Additional file 1. Changes in protein expression in paired neoplasms are shown schematically in Figure 2 and representative staining in Figure 3.

Increased BRCA1 or BRCA2 protein would be expected to increase resistance to platinum therapy. In contrast, decreased MLH1 protein should be associated with platinum resistance. In the paired sets most primary neoplasms (26/31, 84%) had normal MLH1 protein. Of the 33 recurrences occurring in the 26 cases with normal MLH1 protein in the primary, only one had loss of MLH1 protein (3.0%, Figures 2 and 3, and Table S1 in Additional file 1). Neither the primary nor recurrent neoplasm in this pair had MLH1 promoter methylation.

In contrast to MLH1, BRCA1 and BRCA2 expression demonstrated greater variability between paired primary and recurrent neoplasms (Figure 2, Table S1;Additional file 1). In 16 carcinomas recurring after 13 primaries with normal BRCA1 expression, 13(81%) maintained similar protein expression and three (19%) demonstrated decreased BRCA1 expression in the recurrence. In 13 recurrences that followed 11 paired primaries with intermediate BRCA1 expression, four had reduced, seven had increased and two had similar BRCA1 expression in the paired recurrence. In eight recurrences that followed six primary carcinomas with low BRCA1 expression, 2 had similar and six (75%) had increased BRCA1 expression in the recurrence. Overall 13 of 21 (62%) neoplastic pairs with low or intermediate BRCA1 protein expression in the primary had increased BRCA1 protein in the recurrence. In contrast, only eight of 33 (24%) neoplasms with normal or intermediate BRCA1 expression showed reduced BRCA1 expression in the recurrence (p = 0.01). Therefore, increases in BRCA1 expression were more common than decreases in BRCA1 expression following chemotherapy.

BRCA2 protein expression also frequently varied between paired primary and recurrent neoplasms (Figures 2 and 3, Additional file 1). Among primary neoplasms with low or intermediate BRCA2 expression, the paired post-chemotherapy specimen had increased BRCA2 expression in 15/21 cases (71%). In contrast to the observation for BRCA1 protein expression, primary neoplasms with normal or intermediate BRCA2 expression were equally likely to have decreased BRCA2 expression (14/23, 61%).

Excluding those cases with normal BRCA1 and BRCA2 protein expression in the primary, 20 (80%) recurrent carcinomas had increases in either BRCA1 or BRCA2 protein while five (20%) did not have an increase in expression of either protein. Likelihood of a complete response to subsequent chemotherapy was not related to whether increased BRCA1 or BRCA2 protein was identified in the paired recurrence, but wide heterogeneity in treatment precludes assessment of response to specific agents.

### BRCA1, MLH1, and FANCF promoter methylation in primary and recurrent ovarian carcinomas

Methylation was assessed for MLH1, BRCA1 and FANCF in 104 primary and 36 recurrent sporadic carcinomas. Of the primary carcinomas tested for methylation, 11 were obtained after neoadjuvant chemotherapy and 93 were chemotherapy-naive. Results of methylation analysis for BRCA1, FANCF and MLH1 are summarized in Table 3. In no cases were neoplasms methylated at more than one of these genes. Methylation did not vary between primary cases exposed to neoadjuvant chemotherapy or in primary vs. recurrent cases for any gene (Table 3). Ten of the primary and recurrent neoplasms were matched specimens from the same patient collected at two different time points (Table S1; Additional file 1). All ten cases showed concordant methylation between primary and recurrence, and none were methylated at any of the three genes. Reduction of BRCA1 protein was significantly associated with BRCA1 methylation (p = 0.02). However, BRCA1 methylation only accounted for 7/52 (13.5%) of those sporadic carcinomas with low or intermediate BRCA1 expression.

**Table 3:** Methylation of DNA repair genes in primary and recurrent ovarian or peritoneal carcinomas.

	**PROPORTION METHYLATED**		
	**BRCA1**	**MLH1**	**FANCF**
Primary, no chemotherapy	6/91 (6.6%)	3/93 (3.2%)	3/93 (3.2%)
Primary, post neoadjuvant chemo	2/11 (18%)	1/11 (9.1%)	0/10
Recurrent*	2/31 (6.5%)	0/30	0/31 (0%)

**Total (2.2%)**	**10/133 (7.5%)**	**4/134 (3.0%)**	**3/134**

* Ten recurrent carcinomas were paired specimens for which we also evaluated the primary neoplasm. None of these 10 paired cases were methylated at any of the three genes in either the primary or recurrent case. One of these 10 pairs did show loss of MLH1 protein in the recurrent neoplasm, but was not methylated.

All cases with MLH1 methylation demonstrated microsatellite instability at BAT26 (data not shown). MLH1 methylation was associated with loss of protein (p = 0.03), but one methylated case did have apparently normal MLH1 protein expression. MLH1 methylation was more common in endometrioid ovarian carcinomas compared to all other histologies (3/14, 21% versus 1/116, 1%, p = 0.003). FANCF and BRCA1 methylation were not associated with histology.

### Relationship of p53 mutations to protein expression and methylation of DNA repair genes

p53 mutations were assessed by DNA sequencing in all cases tested for methylation and neither BRCA1, MLH1, nor FANCF methylation were associated with p53 mutation status. p53 mutations were more common in sporadic ovarian carcinomas with loss or reduction of BRCA1 expression (Table 4, p = 0.01 Fishers Exact). The majority of p53 mutations were missense mutations occurring in the DNA binding domain (exons 5–8). Null mutations including frameshift, splice site, and nonsense mutations accounted for 9/33 (27%) of p53 mutations.

**Table 4:** p53 mutations and BRCA1 protein level in primary sporadic ovarian carcinomas

	**P53 Mutations**			
**BRCA1 protein Expression**	**Total Tested**	**Missense** **N (%)**	**Null** **N (%)**	**Total P53 mutations**
Low	28	10 (36%)	5 (18%)	15 (54%)
Intermediate	18	8 (44%)	2 (11%)	10 (56%)
Normal	33	6(18%)	2 (6%)	8 (24%)*

Total	79	24 (30%)	9 (11%)	33 (42%)

*p = 0.01 for difference in p53 mutation frequency for cases with normal versus reduction or loss of BRCA1 protein.

### Overall survival and protein expression or promoter methylation

**Figure 4:**
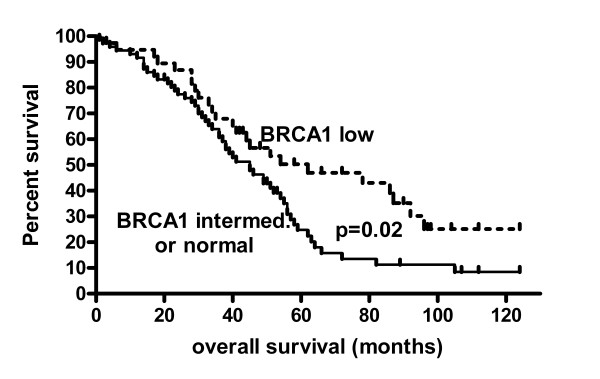
**Overall survival in relation to BRCA1 expression in primary sporadic ovarian carcinomas**. Overall survival was significantly improved in primary ovarian carcinomas (p = 0.02, LogRank test) with low BRCA1 protein expression (median survival 62 months) compared to carcinomas with intermediate or normal BRCA1 expresssion (median survival 45 months).

Individual factors influencing survival in this cohort in univariate analyses were stage (p = 0.01), age (p = 0.03) and optimal cytoreduction to a maximum neoplastic diameter less than 1 cm (p < 0.0001) and low BRCA1 expression (p = 0.02). Low BRCA1 expression in the primary carcinoma was associated with longer survival compared to intermediate or normal BRCA1 expression (median survival 62 months vs. 45 months, p = 0.02 LogRank Test, Hazard Ratio 0.59, 95% confidence interval 0.37–0.93, Figure 4). Low BRCA1 expression remained significantly associated with improved survival in a Cox multi regression model with the covariates age (p = 0.04) or stage (p = 0.04) but was no longer significant when using the co-variate optimal cytoreduction (p = 0.10). Individual factors not related to survival included grade, MLH1 or BRCA2 protein expression, methylation of BRCA1, MLH1 or FANCF genes, or p53 mutation.

### Response to chemotherapy

The likelihood of a complete response to initial chemotherapy was not related to BRCA1 protein levels. Likewise, there was no significant difference in the complete response rate for cases with BRCA1 methylation, MLH1 methylation, or for cases with either BRCA1 or FANCF methylation.

## Discussion

BRCA1 protein loss is common in sporadic epithelial ovarian and peritoneal carcinomas. Low BRCA1 protein expression was associated with a significantly improved overall survival. Our data suggest that somatic loss of BRCA1 favourably influences survival similar to the improved survival in ovarian carcinomas associated with inherited BRCA1 mutations. Two previous studies have examined the relationship between BRCA1 protein expression and prognosis in ovarian carcinomas. Thrall and colleagues evaluated a large number of sporadic advanced stage carcinomas and found that BRCA1 loss was strongly protective for overall survival [29]. In another study of un-selected ovarian carcinoma, Wang and colleagues failed to find an association between BRCA1 protein and prognosis [30]. However, that series evaluated a large number of endometrioid, clear cell, grade 1 and stage I cases, with few high-grade, advanced stage, serous carcinomas, distinctly different from the neoplasms in our study. Indeed, among the 29 stage III and IV cases in Wang's study, there was a trend toward a survival advantage with loss of BRCA1 protein [30]. Our data combined with these two previous studies confirm an improved prognosis for women with advanced ovarian carcinomas with low BRCA1 protein expression.

To our knowledge, this is the first study of BRCA2 protein expression in human ovarian carcinomas. Unlike BRCA1, BRCA2 protein expression was not associated with overall survival. Our data demonstrate that BRCA1 and BRCA2 protein expression are highly concordant in ovarian carcinomas. In both mouse and human, BRCA1 and BRCA2 have nearly identical patterns of message and protein expression in embryonic development, breast morphogenesis, and in many adult tissues, suggesting that the two genes share regulatory networks [31-34]. Similarly, in breast and ovarian carcinoma cell lines, BRCA1 and BRCA2 mRNA are concordantly induced by adriamycin, ionizing radiation, and estrogen [35-37]. Our data suggest that ovarian carcinomas maintain the coordinate regulation of BRCA1 and BRCA2 seen in normal tissues.

We hypothesized that BRCA1 and BRCA2 protein levels would increase after exposure to chemotherapy, thereby mediating increasing platinum resistance during the disease course. Since recurrent ovarian carcinomas are not routinely subjected to biopsy, our cases represent a wide variety of disease time points depending on the clinical indication for biopsy or surgery in a given patient. Consequently, our patients received varied amounts and types of chemotherapy before and after biopsies. An increase in BRCA1 or BRCA2 protein occurred in the majority of recurrent neoplasms that had low or intermediate protein in the paired primary. Increases in BRCA1 protein were significantly more likely than reduction of protein in paired post chemotherapy neoplasms, suggesting a selection for increased BRCA1 expression. In contrast, BRCA2 protein both increased and decreased frequently post chemotherapy. An increasing tendency toward platinum resistance during the typical ovarian carcinoma disease course is a well recognized clinical challenge. Increased BRCA1 and BRCA2 expression could mediate that resistance. However, given the treatment heterogeneity of our cases, we cannot directly relate the alterations in BRCA1 or BRCA2 expression with specific treatment responses.

Our data are consistent with previous reports that BRCA1 promoter methylation occurs in 5–20% of sporadic ovarian carcinomas [20-22,24,25]. Interestingly, we found that promoter methylation only occurs in 15% of those sporadic ovarian carcinomas with low or intermediate BRCA1 expression. Thus, other regulators of BRCA1 expression may be more important than promoter methylation in ovarian carcinomas for both primary and recurrent neoplasms. We found no association between BRCA1 methylation and survival in sporadic ovarian carcinomas, in contrast to a recent small study that demonstrated a survival disadvantage for patients whose neoplasms were methylated at BRCA1 [38]. Our data suggest that BRCA1 protein expression is a better predictor of survival than BRCA1 promoter methylation, not surprising given the relatively small number of cases with BRCA1 methylation.

Low BRCA1 protein expression was associated with an increased likelihood of p53 mutation, low BRCA2 protein expression, and improved survival. Only one previous study of which we are aware has examined the association of somatic BRCA1 alterations and p53 status. In that study, BRCA1 methylation did not correlate with immunohistochemically detected p53 protein expression [22]. However, p53 immunohistochemistry correlates imperfectly with the presence of a somatic mutation, and BRCA1 methylation accounts for only a small fraction of BRCA1 protein loss, so our studies are not equivalent. The majority of breast, ovarian, and peritoneal carcinomas associated with germline mutations in BRCA1 and BRCA2 have p53 mutations, at rates higher than found in their sporadic counterparts [39-42]. Thus, many sporadic ovarian carcinomas have reduction of BRCA1 protein, mutations in p53, and better overall survival, mimicking the phenotype of hereditary BRCA1-associated ovarian carcinoma.

MLH1 is a DNA mismatch repair gene required for sensitivity to platinum compounds. MHL1 is commonly methylated in colorectal and endometrial carcinomas and less commonly in ovarian carcinomas [19,24]. MLH1 methylation mediates platinum resistance in the ovarian carcinoma cell line A2780 [18]. We postulated that chemotherapy exposure could select for neoplastic cells with MLH1 methylation. Indeed, cell-free methylated MLH1 DNA is increased in the plasma of women with ovarian carcinoma at the time of clinical relapse [43]. Increased methylation of MLH1 in recurrent ovarian carcinoma has been proposed as a rationale for clinical trials of demethylating agents alone or in combination with chemotherapy. We found no association between MLH1 protein and overall survival or response to chemotherapy, and we rarely observed (3.8%) loss of MLH1 protein in paired neoplasms following chemotherapy exposure. Our data contrast with those of two groups who both reported a significant decrease in MLH1 protein expression in paired ovarian carcinomas following platinum chemotherapy [44,45]. But surprisingly, Fink and colleagues found that MLH1 protein expression correlated inversely with treatment response, opposite to the expected association [44]. We used a traditional cut-off to define MLH1 protein deficiency at 10% of cells while the other two studies used a continuous scoring system. However, even when we re-evaluated our data with a continuous scoring system, we still found that significant decrease in MLH1 protein expression was rare (data not shown). The discrepancies between these studies and ours may stem from the clinical heterogeneity of cases for which paired primary and recurrent ovarian carcinomas are available. Finally, we did not find MLH1 promoter methylation more frequently in either recurrent neoplasms or in primary neoplasms exposed to neoadjuvant chemotherapy, nor was MLH1 methylation identified in the neoplastic pair with reduced MLH1 expression in the recurrence. Consequently, our data do not support a major role for *in vivo *epigenetic alteration of MLH1 expression in the development of clinical platinum resistance in sporadic ovarian carcinomas.

FANCF may also mediate platinum sensitivity. FANCF promoter methylation confers cisplatin sensitivity to cell lines and was hypothesized to be a mediator of *in vivo *platinum sensitivity[23]. In order to mediate resistance, a neoplasm should be initially methylated and then lose methylation in recurrent disease. However, our findings indicate that FANCF methylation occurs rarely in primary ovarian carcinomas (<5% of cases), and is therefore not likely to be a major clinical mediator of platinum sensitivity.

## Conclusion

Low BRCA1 protein expression in sporadic ovarian carcinomas is associated with a favourable survival and an increased rate of p53 mutations. BRCA1 and BRCA2 proteins are concordantly expressed in sporadic ovarian and peritoneal carcinomas. Increases in BRCA1 and BRCA2 protein expression are common in recurrent sporadic ovarian carcinomas, but the mechanism(s) responsible for these expression differences are unknown. Alteration in methylation of the promoters of BRCA1, MLH1, and FANCF do not seem to commonly mediate clinical chemoresistance in women with ovarian carcinomas.

## Methods

### Specimens

Patients consented to have tissue collected and to provide blood samples using protocols approved by the Human Subjects Division of the University of Washington Institutional Review Board. Paraffin blocks were obtained from pathology archives. Tissues were obtained at the time of surgery and snap frozen in liquid nitrogen. Tissue sections were stained with hematoxylin and eosin and reviewed for neoplastic cell percentage prior to DNA extraction. DNA was extracted using the Stratagene^® ^kit according to the manufacturer's instructions. Normal DNA was extracted from peripheral white blood cells or from normal tissue after histological review excluded contamination with neoplasm. Family history was considered strong if the patient had a known BRCA1 or BRCA2 mutation, personal history of breast carcinoma, any relative with ovarian carcinoma, a first degree relative with premenopausal breast carcinoma, or two relatives of any degree with breast carcinoma at any age. Cases without strong family histories were considered sporadic, while those with suggestive family histories were considered familial and excluded.

### Immunohistochemical staining for MLH1, BRCA1 and BRCA2

BRCA1 protein was detected in formalin fixed paraffin sections using the mouse monoclonal antibody MS110 (previously called Ab-1, Oncogene Research Products) as previously described [46]. The MS110 antibody recognizes an amino terminal epitope in BRCA1 (amino acid residues 89–222). BRCA2 was detected using the H-300 rabbit polyclonal antibody (Santa Cruz Biotech) that recognizes amino acid residues 2520–2819 of human BRCA2. MLH1 was detected using the mouse monoclonal antibody G168-728 (BD Pharmingen). Briefly, paraffin sections were deparaffinized, re-hydrated, and treated with steam heat for 20 minutes using antigen target retrieval solution (DAKO). Endogenous peroxidase activity was quenched by treatment with 3% H_2_O_2 _for 5 minutes. Sections were washed with PBS and non-specific binding was blocked by treatment for three hours in 2% bovine serum albumin in PBS. Primary antibody was diluted in PBS (MS110 at 1:250 dilution, H-300 at 1:100 dilution, and G168-728 at 1:80) and applied to sections for 14–16 hours at 4° (MS110 and H-300) or 40 minutes at room temperature (G168-728). Secondary antibody and streptavidin biotin-peroxidase were from Universal Large Volume LSAB+, Peroxidase kit (DAKO) and were each applied for 30 minutes at room temperature. DAB (3,3'diaminobenzidine)-nickel or DAB chromagen (DAKO) was used to visualize antibody complexes. Sections were counterstained with methyl green or hematoxylin. Staining of inflammatory cells served as a positive internal control for all antibodies.

In order to demonstrate the specificity of the BRCA2 H-300 antibody we tested our protocol on a primary tumor with a BRCA2 mutation that results in protein truncation prior to the epitope recognized by H-300. This carcinoma has no detectable wildtype sequence. We also evaluated BRCA2 on its paired recurrence with a known genetic reversion and presumed normal expression of BRCA2[12]. Protein expression was absent in the primary carcinoma and strongly present in all recurrent neoplastic nuclei consistent with the DNA sequence information (data not shown).

Controversy exists regarding the meaning and specificity of cytoplasmic staining for BRCA1 and BRCA2. Proteins encoded by aberrantly spliced isoforms have been shown to localize to the cytoplasm for both BRCA1 and BRCA2 [47-49]. The MS110 antibody has better specificity than other anti-BRCA1 antibodies and has an almost exclusively nuclear staining pattern[46]. We observed exclusively nuclear BRCA1 protein staining in both normal and neoplastic tissues. We observed both nuclear and cytoplasmic BRCA2 staining in neoplastic cells, but scored only nuclear staining as likely to be relevant to intact DNA repair. BRCA1 and BRCA2 protein was scored as previously described: "low" if fewer than 10% of neoplastic cells had nuclear staining and "intermediate" if 10% to 30% of neoplastic cells had nuclear staining and normal if greater than 30% of neoplastic cells had nuclear staining [46]. MLH1 protein expression was scored as loss (<10% of cells positive) or normal (>10% of cells positive) as is done routinely for clinical purposes. MLH1 staining was exclusively nuclear. For the purpose of comparing protein expression in paired primary and recurrent neoplasms, the second recurrence in those seven cases with more than one recurrence was compared with the primary neoplasms and counted as a separate case.

### Methylation specific PCR

Carcinomas were evaluated for BRCA1, MLH1 and FANCF promoter methylation as previously described [21,23,50]. All methylation specific PCR assays included a positive control (in vitro methylated DNA, Invitrogen) and a negative (water) control. Positive assays were repeated at least once. Sodium bisulfite treated DNA was amplified with primers specific for either methylated or unmethylated treated substrate. PCR products were electrophoresed on 3% agarose gels or 5% acrylamide gels and stained with ethidium bromide.

### p53 mutation detection

DNA was amplified in separate PCR reactions for all p53 coding exons (2–11) and flanking regulatory regions. Primer sequences and PCR conditions are available from the authors on request. PCR products were purified and sequenced using Big Dye Terminator chemistry (Perkin-Elmer, Boston, MA), and run on an ABI 3100 DNA sequencer (Applied Biosytstems, Foster City, CA). Sequencing data were analyzed using Sequencher software (Gene Codes Corporation, Ann Arbor MI). Mutations were confirmed with a separate sequencing reaction and were compared to the UMP TP53 mutation database at .

### Statistics and Survival

Two by two comparisons were evaluated with the Fisher's exact tests and larger contingency tables were evaluated with Chi Square. Two-tailed p values were generated with InStat (Graphpad Software, San Diego, CA). All women received primary therapy containing a taxane and platinum agent. Overall survival was calculated from the date of diagnosis until death or last follow-up. Survival curves were generated according to the Kaplan-Meier method using Prism (Graphpad Software). Cox regression analysis was performed using JMP8 (SAS, Cary, NC). Differences between survival curves were tested with the Log-rank method. Multi-variate analysis was performed using Cox proportional hazards modelling using JMP8.

## Competing interests

The authors declare that they have no competing interests.

## Authors' contributions

EMS performed molecular assays and drafted the manuscript. RG performed protein expression assays. RG reviewed specimen pathology. PW co-wrote the manuscript and contributed to the study design. TW designed methylation assays. TT designed the FANCF assays and edited the manuscript. BG contributed to sample acquisition and study design. All authors read and approved the final manuscript.

## Supplementary Material

Additional file 1 **Table S1**. Clinical and molecular data for all paired primary and recurrent carcinomas Click here for file

## References

[B1] Howlett NG, Taniguchi T, Olson S, Cox B, Waisfisz Q, De Die-Smulders C, Persky N, Grompe M, Joenje H, Pals G (2002). Biallelic inactivation of BRCA2 in Fanconi anemia. Science.

[B2] Wang W (2007). Emergence of a DNA-damage response network consisting of Fanconi anaemia and BRCA proteins. Nat Rev Genet.

[B3] Husain A, He G, Venkatraman ES, Spriggs DR (1998). BRCA1 up-regulation is associated with repair-mediated resistance to cis-diamminedichloroplatinum(II). Cancer Res.

[B4] Quinn JE, Kennedy RD, Mullan PB, Gilmore PM, Carty M, Johnston PG, Harkin DP (2003). BRCA1 functions as a differential modulator of chemotherapy-induced apoptosis. Cancer Res.

[B5] Bhattacharyya A, Ear US, Koller BH, Weichselbaum RR, Bishop DK (2000). The breast cancer susceptibility gene BRCA1 is required for subnuclear assembly of Rad51 and survival following treatment with the DNA cross-linking agent cisplatin. J Biol Chem.

[B6] Lafarge S, Sylvain V, Ferrara M, Bignon YJ (2001). Inhibition of BRCA1 leads to increased chemoresistance to microtubule-interfering agents, an effect that involves the JNK pathway. Oncogene.

[B7] Zhou C, Smith JL, Liu J (2003). Role of BRCA1 in cellular resistance to paclitaxel and ionizing radiation in an ovarian cancer cell line carrying a defective BRCA1. Oncogene.

[B8] Boyd J, Sonoda Y, Federici MG, Bogomolniy F, Rhei E, Maresco DL, Saigo PE, Almadrones LA, Barakat RR, Brown CL (2000). Clinicopathologic features of BRCA-linked and sporadic ovarian cancer. Jama.

[B9] Cass I, Baldwin RL, Varkey T, Moslehi R, Narod SA, Karlan BY (2003). Improved survival in women with BRCA-associated ovarian carcinoma. Cancer.

[B10] Chetrit A, Hirsh-Yechezkel G, Ben-David Y, Lubin F, Friedman E, Sadetzki S (2008). Effect of BRCA1/2 mutations on long-term survival of patients with invasive ovarian cancer: the national Israeli study of ovarian cancer. J Clin Oncol.

[B11] Edwards SL, Brough R, Lord CJ, Natrajan R, Vatcheva R, Levine DA, Boyd J, Reis-Filho JS, Ashworth A (2008). Resistance to therapy caused by intragenic deletion in BRCA2. Nature.

[B12] Sakai W, Swisher EM, Karlan BY, Agarwal MK, Higgins J, Friedman C, Villegas E, Jacquemont C, Farrugia DJ, Couch FJ (2008). Secondary mutations as a mechanism of cisplatin resistance in BRCA2-mutated cancers. Nature.

[B13] Swisher EM, Sakai W, Karlan BY, Wurz K, Urban N, Taniguchi T (2008). Secondary BRCA1 mutations in BRCA1-mutated ovarian carcinomas with platinum resistance. Cancer Res.

[B14] Berchuck A, Heron KA, Carney ME, Lancaster JM, Fraser EG, Vinson VL, Deffenbaugh AM, Miron A, Marks JR, Futreal PA, Frank TS (1998). Frequency of germline and somatic BRCA1 mutations in ovarian cancer. Clin Cancer Res.

[B15] Foster KA, Harrington P, Kerr J, Russell P, DiCioccio RA, Scott IV, Jacobs I, Chenevix-Trench G, Ponder BA, Gayther SA (1996). Somatic and germline mutations of the BRCA2 gene in sporadic ovarian cancer. Cancer Res.

[B16] Takahashi H, Chiu HC, Bandera CA, Behbakht K, Liu PC, Couch FJ, Weber BL, LiVolsi VA, Furusato M, Rebane BA (1996). Mutations of the BRCA2 gene in ovarian carcinomas. Cancer Res.

[B17] Takahashi H, Behbakht K, McGovern PE, Chiu HC, Couch FJ, Weber BL, Friedman LS, King MC, Furusato M, LiVolsi VA (1995). Mutation analysis of the BRCA1 gene in ovarian cancers. Cancer Res.

[B18] Strathdee G, MacKean MJ, Illand M, Brown R (1999). A role for methylation of the hMLH1 promoter in loss of hMLH1 expression and drug resistance in ovarian cancer. Oncogene.

[B19] Willner J, Wurz K, Allison KH, Galic V, Garcia RL, Goff BA, Swisher EM (2007). Alternate molecular genetic pathways in ovarian carcinomas of common histological types. Hum Pathol.

[B20] Catteau A, Harris WH, Xu CF, Solomon E (1999). Methylation of the BRCA1 promoter region in sporadic breast and ovarian cancer: correlation with disease characteristics. Oncogene.

[B21] Esteller M, Silva JM, Dominguez G, Bonilla F, Matias-Guiu X, Lerma E, Bussaglia E, Prat J, Harkes IC, Repasky EA (2000). Promoter hypermethylation and BRCA1 inactivation in sporadic breast and ovarian tumors. J Natl Cancer Inst.

[B22] Baldwin RL, Nemeth E, Tran H, Shvartsman H, Cass I, Narod S, Karlan BY (2000). BRCA1 promoter region hypermethylation in ovarian carcinoma: a population-based study. Cancer Res.

[B23] Taniguchi T, Tischkowitz M, Ameziane N, Hodgson SV, Mathew CG, Joenje H, Mok SC, D'Andrea AD (2003). Disruption of the Fanconi anemia-BRCA pathway in cisplatin-sensitive ovarian tumors. Nat Med.

[B24] Strathdee G, Appleton K, Illand M, Millan DW, Sargent J, Paul J, Brown R (2001). Primary ovarian carcinomas display multiple methylator phenotypes involving known tumor suppressor genes. Am J Pathol.

[B25] Teodoridis JM, Hall J, Marsh S, Kannall HD, Smyth C, Curto J, Siddiqui N, Gabra H, McLeod HL, Strathdee G, Brown R (2005). CpG island methylation of DNA damage response genes in advanced ovarian cancer. Cancer Res.

[B26] Gras E, Cortes J, Diez O, Alonso C, Matias-Guiu X, Baiget M, Prat J (2001). Loss of heterozygosity on chromosome 13q12-q14, BRCA-2 mutations and lack of BRCA-2 promoter hypermethylation in sporadic epithelial ovarian tumors. Cancer.

[B27] Hilton JL, Geisler JP, Rathe JA, Hattermann-Zogg MA, DeYoung B, Buller RE (2002). Inactivation of BRCA1 and BRCA2 in ovarian cancer. J Natl Cancer Inst.

[B28] Brown R, Hirst GL, Gallagher WM, McIlwrath AJ, Margison GP, Zee AG van der, Anthoney DA (1997). hMLH1 expression and cellular responses of ovarian tumour cells to treatment with cytotoxic anticancer agents. Oncogene.

[B29] Thrall M, Gallion HH, Kryscio R, Kapali M, Armstrong DK, DeLoia JA (2006). BRCA1 expression in a large series of sporadic ovarian carcinomas: a Gynecologic Oncology Group study. Int J Gynecol Cancer.

[B30] Wang C, Horiuchi A, Imai T, Ohira S, Itoh K, Nikaido T, Katsuyama Y, Konishi I (2004). Expression of BRCA1 protein in benign, borderline, and malignant epithelial ovarian neoplasms and its relationship to methylation and allelic loss of the BRCA1 gene. J Pathol.

[B31] Blackshear PE, Goldsworthy SM, Foley JF, McAllister KA, Bennett LM, Collins NK, Bunch DO, Brown P, Wiseman RW, Davis BJ (1998). Brca1 and Brca2 expression patterns in mitotic and meiotic cells of mice. Oncogene.

[B32] Rajan JV, Wang M, Marquis ST, Chodosh LA (1996). Brca2 is coordinately regulated with Brca1 during proliferation and differentiation in mammary epithelial cells. Proc Natl Acad Sci USA.

[B33] Bernard-Gallon DJ, Peffault de Latour M, Hizel C, Vissac C, Cure H, Pezet D, Dechelotte PJ, Chipponi J, Chassagne J, Bignon YJ (2001). Localization of human BRCA1 and BRCA2 in non-inherited colorectal carcinomas and matched normal mucosas. Anticancer Res.

[B34] Rajan JV, Marquis ST, Gardner HP, Chodosh LA (1997). Developmental expression of Brca2 colocalizes with Brca1 and is associated with proliferation and differentiation in multiple tissues. Dev Biol.

[B35] Andres JL, Fan S, Turkel GJ, Wang JA, Twu NF, Yuan RQ, Lamszus K, Goldberg ID, Rosen EM (1998). Regulation of BRCA1 and BRCA2 expression in human breast cancer cells by DNA-damaging agents. Oncogene.

[B36] Fan S, Twu NF, Wang JA, Yuan RQ, Andres J, Goldberg ID, Rosen EM (1998). Down-regulation of BRCA1 and BRCA2 in human ovarian cancer cells exposed to adriamycin and ultraviolet radiation. Int J Cancer.

[B37] Spillman MA, Bowcock AM (1996). BRCA1 and BRCA2 mRNA levels are coordinately elevated in human breast cancer cells in response to estrogen. Oncogene.

[B38] Chiang JW, Karlan BY, Cass L, Baldwin RL (2006). BRCA1 promoter methylation predicts adverse ovarian cancer prognosis. Gynecol Oncol.

[B39] Rhei E, Bogomolniy F, Federici MG, Maresco DL, Offit K, Robson ME, Saigo PE, Boyd J (1998). Molecular genetic characterization of BRCA1- and BRCA2-linked hereditary ovarian cancers. Cancer Res.

[B40] Ramus SJ, Bobrow LG, Pharoah PD, Finnigan DS, Fishman A, Altaras M, Harrington PA, Gayther SA, Ponder BA, Friedman LS (1999). Increased frequency of TP53 mutations in BRCA1 and BRCA2 ovarian tumours. Genes Chromosomes Cancer.

[B41] Crook T, Crossland S, Crompton MR, Osin P, Gusterson BA (1997). p53 mutations in BRCA1-associated familial breast cancer. Lancet.

[B42] Greenblatt MS, Chappuis PO, Bond JP, Hamel N, Foulkes WD (2001). TP53 mutations in breast cancer associated with BRCA1 or BRCA2 germ-line mutations: distinctive spectrum and structural distribution. Cancer Res.

[B43] Gifford G, Paul J, Vasey PA, Kaye SB, Brown R (2004). The acquisition of hMLH1 methylation in plasma DNA after chemotherapy predicts poor survival for ovarian cancer patients. Clin Cancer Res.

[B44] Fink D, Nebel S, Norris PS, Baergen RN, Wilczynski SP, Costa MJ, Haas M, Cannistra SA, Howell SB (1998). Enrichment for DNA mismatch repair-deficient cells during treatment with cisplatin. Int J Cancer.

[B45] Samimi G, Fink D, Varki NM, Husain A, Hoskins WJ, Alberts DS, Howell SB (2000). Analysis of MLH1 and MSH2 expression in ovarian cancer before and after platinum drug-based chemotherapy. Clin Cancer Res.

[B46] Wilson CA, Ramos L, Villasenor MR, Anders KH, Press MF, Clarke K, Karlan B, Chen JJ, Scully R, Livingston D (1999). Localization of human BRCA1 and its loss in high-grade, non-inherited breast carcinomas. Nat Genet.

[B47] Thakur S, Zhang HB, Peng Y, Le H, Carroll B, Ward T, Yao J, Farid LM, Couch FJ, Wilson RB, Weber BL (1997). Localization of BRCA1 and a splice variant identifies the nuclear localization signal. Mol Cell Biol.

[B48] Wilson CA, Payton MN, Elliott GS, Buaas FW, Cajulis EE, Grosshans D, Ramos L, Reese DM, Slamon DJ, Calzone FJ (1997). Differential subcellular localization, expression and biological toxicity of BRCA1 and the splice variant BRCA1-delta11b. Oncogene.

[B49] Spain BH, Larson CJ, Shihabuddin LS, Gage FH, Verma IM (1999). Truncated BRCA2 is cytoplasmic: implications for cancer-linked mutations. Proc Natl Acad Sci USA.

[B50] Herman JG, Umar A, Polyak K, Graff JR, Ahuja N, Issa JP, Markowitz S, Willson JK, Hamilton SR, Kinzler KW (1998). Incidence and functional consequences of hMLH1 promoter hypermethylation in colorectal carcinoma. Proc Natl Acad Sci USA.

